# Efficacy of *Sida pilosa* Retz aqueous extract against *Schistosoma mansoni* – induced granulomatous inflammation in the liver and the intestine of mice: histomorphometry and gastrointestinal motility evaluation

**DOI:** 10.1186/s12906-018-2318-2

**Published:** 2018-09-06

**Authors:** Hermine Boukeng Jatsa, Ulrich Membe Femoe, Joseph Njiaza, Daniel Simplice Tombe Tombe, Lohik Nguegan Mbolang, Emilienne Tienga Nkondo, Louis-Albert Tchuem Tchuente, Théophile Dimo, Pierre Kamtchouing

**Affiliations:** 1 Laboratory of Animal Physiology, Department of Animal Biology and Physiology, Faculty of Science, University of Yaoundé I, P.O. Box 812, Yaoundé, Cameroon; 2 Laboratory of Animal Biology, Department of Animal Biology and Physiology, Faculty of Science, University of Yaoundé I, P.O. Box 812, Yaoundé, Cameroon; 3 Centre for Schistosomiasis and Parasitology, P.O. Box 7244, Yaoundé, Cameroon

**Keywords:** Inflammation, *Sida pilosa*, Histomorphometry, Gastrointestinal motility, *Schistosoma mansoni*

## Abstract

**Background:**

The macerate of *Sida pilosa* aerial parts is used empirically for the treatment of intestinal helminthiasis. Previous studies have shown that *Sida pilosa* aqueous extract (SpAE) has schistosomicidal, antioxidant, anti-inflammatory and anti-fibrotic activities in *Schistosoma mansoni* infection. This study was designed to evaluate the effect of SpAE on the granulomatous inflammation induced by *S. mansoni* in the liver and the intestine of mice by histomorphometry; as well as on the gastrointestinal motility.

**Methods:**

To study the effect of SpAE on the liver and intestine histomorphometry and on the gastrointestinal motility, SpAE was administered at 200 mg/kg per os to *S. mansoni*-infected mice for 4 weeks. Praziquantel was used as reference drug. Prior to carrying out sacrifice, a batch of mice was subjected to gastrointestinal transit evaluation with 3% charcoal meal. After sacrifying another batch of mice, we performed histological and morphometric analyses of the liver and the ileum. We measured the following: total proteins, transaminases, malondialdehyde, nitrites, superoxide dismutase, catalase and reduced glutathione. The effect of SpAE (4, 8, 16 and 32 mg/mL) on the ileum contractile activity was evaluated either in the absence or in the presence of pharmacological blockers.

**Results:**

SpAE induced a significant reduction of hepatosplenomegaly and intestine enlargement. The number of granulomas was reduced by 52.82% in the liver and 52.79% in the intestine, whereas the volume of hepatic granulomas decreased by 48.76% after SpAE treatment. SpAE also reduced (*p* < 0.001) the ileal muscular layer thickness. The levels of total proteins, transaminases, malondialdehyde, nitrites, superoxide dismutase, catalase and reduced glutathione were restored after treatment of infected mice with SpAE. A normalization of the gastrointestinal transit was also recorded after SpAE treatment. The effect of SpAE on intestinal motility was mediated via intracellular and extracellular calcium mobilization.

**Conclusion:**

Our findings provide evidence that SpAE improves granulomatous inflammation induced by *S. mansoni* both in the liver and in the intestine, as well as it re-establishes normal gastrointestinal transit. SpAE may be used for the development of alternative medicine against *S. mansoni* infection.

## Background

Schistosomiasis is the most prevalent and debilitating neglected diseases in the tropics and subtropics. It is associated with severe morbidity and mortality. Approximately 7 weeks after the initial transcutaneous infection, schistosomes migrate to the host hepatic portal system and egg production starts in the tributaries of the inferior mesenteric vein [1]. Adult *Schistosoma mansoni* worms reside in the mesenteric veins for years where they lay eggs and release antigens, which eventually lead to death. The antigen-laden eggs are taken with the blood flow and embolize in the liver, causing granulomatous hepatitis leading to fibrosis and portal hypertension [2]. In Latin America, Africa, and Asia, schistosomiasis is one of the most important cause of noncirrhotic portal hypertension [3]. *S. mansoni* eggs induced a granulomatous reaction associated with an overproduction of reactive oxygen and nitrogen species, as well as an impairment of the antioxidant defence [4–8]. During *S. mansoni* life cycle in humans, a part of the eggs reaches the terminal ileum and the colon to be excreted along with the feces, while the other part remains entrapped within the gut wall. In that location, antigenic substances released by the eggs initiate granulomatous inflammation, resulting in intestinal lesions [1]. Eighty-five percent of *S. mansoni*-infected individuals suffer from motility-related gastrointestinal symptoms such as diarrhea, anorexia, nausea, abdominal cramps and constipation [2, 9, 10]. These clinical manifestations may result from gastrointestinal neuromuscular dysfunction induced by the inflammatory cells infiltration in the gut wall [1]. The effect of granulomatous inflammation on gastrointestinal motility in *S. mansoni* infection has been demonstrated. Gastrointestinal transit was inhibited during chronic infection and was associated with inflammation of the intestine wall and with an increase of in vitro contractility of longitudinal muscle strips of the ileum [1, 2, 11, 12]. A schistosomicidal treatment that could prevent or suppress the granulomatous inflammation, could, therefore, avert the development of severe disease [13].

For two decades, praziquantel has remained the only drug against all human schistosome species. Poor cure rates and reduced susceptibility of this drug among some isolates of *S. mansoni* has been demonstrated [14]. This raised concerns about the search and development of complementary and/or alternative new schistosomicidal drugs that are both effective and safe. The value of many plant species used in traditional medicine to treat schistosomiasis is increasingly being recognized. *Sida pilosa* Retz (Malvaceae), is a creeping plant founded mainly on the outskirts of dwelling areas and on wastelands. In Cameroon, the macerate of the plant aerial parts is used for the treatment of intestinal helminthiasis [15]. Pharmacological and toxicological studies have demonstrated the schistosomicidal activity and the safety of *S. pilosa* aqueous extract. Phytochemical screening has revealed the presence of alkaloids, phenols, flavonoids, tannins, and terpenoids in the extract [16]. Fractionation of *S. pilosa* aqueous extract was performed and the HPLC-MS analysis of its *n*-butanol fraction revealed the presence of two indoloquinoline alkaloids. Moreover, this fraction displayed good in vitro activity against *S. mansoni* [17]. However, in vivo studies on the evaluation of the schistosomicidal, antioxidant, anti-inflammatory and anti-fibrotic activities of *S. pilosa* aqueous extract and its *n*-butanol fraction has demonstrated that the aqueous extract was more active than the fraction [7]. The spasmogenic effect of *Sida veronicaefolia* (syn. *Sida pilosa*) on ileal and uterine smooth muscle has been also reported [18, 19]. The present study was performed to evaluate the effect of *S. pilosa* aqueous extract on the granulomatous inflammation in the liver and the intestine by histomorphometry as well as on the gastrointestinal transit after *S. mansoni* infection. To verify the hypothesis that *S. pilosa* aqueous extract could contribute to maintaining a normal gastrointestinal transit, its effect on the contractile activity of the ileum was investigated.

## Methods

### Plant extraction

*Sida pilosa* was collected in March 2009 at Leboudi 2 near Yaoundé city in the Centre region of Cameroon. The plant was identified by Pr. Louis Zapfack by comparison against the specimen Lejoly n° 86/399 in the National Herbarium of Yaoundé, Cameroon, were a voucher specimen was deposited and conserved under the number 53202/HNC.

*S. pilosa* aerial parts were dried in the shade, powdered and mixed with water (100 g/1 L) for 24 h of maceration at room temperature. The solution was filtered, froze at − 20 °C and then lyophilized to give the crude aqueous extract of *S. pilosa* (SpAE), with a recovery rate of 13.61%. SpAE were dissolved in distilled water upon use.

### Animals

Forty-eight (48) eight-week-old BALB/c female mice, weighing 25–30 g, from the animal house of the “Centre for Schistosomiasis and Parasitology” of Yaoundé – Cameroon, and twenty (20) twelve-week-old male Wistar rats weighing 215–230 g, from the animal house of the “Laboratory of Animal Physiology of University of Yaoundé I” were used for this study. They were housed in polypropylene cages under natural 12 h light/12 h dark cycles, temperature maintained between 22 and 25 °C and humidity between 60 and 70%. Mice and rats were fed with rodents’ diet and water ad libitum.

All procedures in this study comply with the principles of laboratory animal use and care of the “European Community” guidelines (EEC Directive 2010/63/EEC) and were approved by the Animal Ethical Committee of the Laboratory of Animal Physiology of the Faculty of Sciences, University of Yaoundé I–Cameroon.

### Gastrointestinal transit and histological study

#### Experimental design

Mice were individually infected with 60 *S. mansoni* cercariae (Cameroonian strain) freshly released from *Biomphalaria pfeifferi* [6]. They were randomly divided into four groups of twelve (12) animals each. Mice were treated per os as follow:

- Group UIC: uninfected-untreated mice receiving the vehicle only (H_2_O).
- Group IC: infected-untreated mice receiving the vehicle only (H_2_O).
- Group PZQ: infected mice receiving praziquantel at the dose of 100 mg/kg for 5 consecutive days followed by distilled water till the end of the experiment.
- SpAE 200: infected mice receiving *S. pilosa* aqueous extract at 200 mg/kg 6 days per week for 4 weeks, starting from the sixth week post-infection.

The dose of 200 mg/kg of SpAE was chosen for this study given that, in our previous study, it was one of the doses to display the best antioxidant, anti-inflammatory and anti-fibrotic activities [7].

After treatment, each group was divided into two (02) sub-groups of six (06) animals each. Mice of the first’s sub-groups were involved in the evaluation of the intestinal transit before their sacrifice on the tenth weeks’ post-infection. Mice of the second’s sub-groups were sacrificed by cervical dislocation on the tenth weeks’ post-infection for histological study and evaluation of some biochemical parameters. The liver, intestine and spleen weights were also determined.

#### Evaluation of the gastrointestinal transit

Prior to the experiment, mice of the first batch of sub-groups had been starved for 18 h. However, they were allowed free access to water. Then, each mouse received per os 0.3 mL of 3% active charcoal suspension in saline as a charcoal meal. After 15 min, mice were sacrificed by cervical dislocation and the abdomens were immediately opened to excise the whole small intestine. We measured the length of the small intestine (from the pylorus sphincter to ileo-cæcal sphincter) as well as the distance between the pylorus region and the front of the charcoal meal. The intestinal transit ratio was calculated using the percentage of the small intestine length in which the charcoal had traveled [20].

#### Morphometric analysis of the liver and the ileum

After the sacrifice, the liver, intestine, and spleens were removed from each mouse, weighed and their relative weights (g of organ / 100 g of body weight) calculated.

For each animal, the right lobe of the liver and the distal portion of the ileum were immersed in 10% formaldehyde prepared in phosphate-buffered saline (PBS). After dehydration in successive baths of ethanol (50 °C –100 °C) and xylene, we embedded tissue samples in paraffin blocks and cut them into 5 μm-thick sections. In order to evaluate the number and volume of granulomas in the liver and in the intestine, as well as the thickness of the ileal muscular layer, sections of the liver and transverse sections of the ileum were stained with hematoxylin and eosin (H&E).

The stained sections were captured with an X10 objective at an X10 ocular magnification with a digital camera (DCM35; Scopotek®, China) and analyzed with a morphometric system Image J1.32 software. We counted the number of microscopic fields and granulomas on each liver or intestine section. Results were expressed as the number of granulomas / 10 microscopic fields.

For the measurement of liver granulomas, only those containing a single central egg with miracidia were selected for each mouse. Granulomas in which it was possible to observe more than one egg or granulomas in which the egg was not visible or destroyed were excluded from the analysis. All granulomas assuming the criteria were measured: the largest diameter and the one perpendicular were averaged. Assuming that a granuloma has a spherical shape, we used the following formula to calculate the volume of each granuloma: Volume of sphere = 4/3 π R^3^ (the radius R was obtained by dividing the main diameter of the lesion by two). We then calculated the mean granuloma volume for each group [13, 21].

We measured the thickness of the muscular layer of the ileum on each transverse section at least at ten different locations. Then we evaluated the mean value in each animal. To avoid bias, we measured muscular layer thickness only on granuloma-free portions [1].

#### Evaluation of some biochemical parameters

We measured transaminases activities in the plasma after sacrificing the mice. Blood from each mouse was then collected from the retro-orbital venous plexus in EDTA tube and centrifuged at 3500 rpm for 15 min. Plasma obtained was stored at − 70 °C until analysis. Using the method of Biuret, we determined total proteins level. The amount of protein was calculated from a standard curve using serial concentration of bovine serum albumin. We assessed the activities of alanine aminotransferase (ALT) and aspartate aminotransferase (AST) with the method described by Reitman and Frankel [22] using kits from BIOLABO (Les Hautes Rives, France).

We evaluated oxidative stress in the liver by measuring some biomarkers. The left lobe of the liver (0.4 g) was homogenized in 2 ml of a Tris-HCl 50 mmol buffer, pH 7.4. The homogenate (20% *w*/*v*) was centrifuged at 3000 rpm for 25 min at 4 °C. The supernatant was collected and stored at − 70 °C until assayed. Lipid peroxides were measured as thiobarbituric acid reactive substances. The product of the reaction between malondialdehyde and thiobarbituric acid was estimated as described by Wilbur et al. [23]. Nitrites concentration was determined using the Griess reagent [24]. Superoxide dismutase (SOD) activity was estimated according to the protocol described by Misra et Fridovish [25]. Catalase activity was assayed following the protocol described by Sinha [26] and reduced glutathione (GSH) was assayed following the method described by Ellman [27].

### Contractile activity of the ileum

In view of evaluating the effect of *S. pilosa* aqueous extract (SpAE) on the intestine contractile activity, an in vitro study was conducted on segments of rat ileum incubated with different concentrations of SpAE.

#### Tissue preparation and mounting

Twenty (20) normal male rats were acclimated for 5 days before used. Rats were starved for food for 24 h but allowed free access to water. They were sacrificed by cervical dislocation without anesthesia to prevent influence on the contractile response of the tissue [28]. After a laparotomy incision, 2 or 3 small segments of the ileum (2 cm long) were excised and gently cleaned of fat and adherent connective tissues. Segments of ileum were maintained in Petri dishes containing Tyrode’s solution with the following composition in mM: NaCl 136.9, CaCl_2_ 1.8; KCl 2.7; MgSO_4_ 1.1, NaHCO_3_ 11.9; NaH_2_PO_4_ 0.42 and glucose 5.6 [29]. Ileum preparations were subsequently mounted in a 20 mL organ bath containing Tyrode’s solution maintained at 37 °C and constantly aerated with carbogen gas (5% CO_2_ and 95% O_2_) to maintain the pH at 7.4. The lower end of an ileum segment was attached to a fixed hook at the bottom of the organ bath, and the upper end was connected with a steel hook to an isotonic transducer combined with a multichannel recorder, Biopac Lab Systems MP 35 (BIOPAC Systems, Santa Barbara, USA). Therefore, we could continuously record mechanical activity monitored by a computer. The tissue was allowed to equilibrate for a period of 60 min under a resting tension of 1 g. During this period the tissue was washed every 15 min [28].

#### Effects of *S. pilosa* aqueous extract on ileum spontaneous contractions

*S. pilosa* aqueous extract (SpAE) was tested on isolated rat ileum spontaneous contractions in concentrations of 4, 8, 16 and 32 mg/mL. Each concentration was added to the bath solution and its effects were recorded for 20 min. Between two administrations of the extract at different concentrations, the tissue was washed three to four times with fresh Tyrode’s solution and allowed to fully recover (40–50 min) before any further administration. Then, we constructed a time–response curve of each concentration of the extract [28]. Similarly, we examined the effects of acetylcholine (10^− 7^ M), a standard spasmogenic drug that served as positive control. We treated the negative control preparations with the solvent of the extract (distilled water) in a volume equivalent to that of the maximal concentration of the extract. This experiment was repeated five times. The effects of the extract were evaluated on the amplitude, tonus, and frequency of spontaneous contractions.

In order to evaluate the concentration-response effect of the extract and to determine it median effective concentration (EC_50_), increasing concentrations of SpAE (2, 4, 8, 16 and 32 mg/mL) were cumulatively added in the organ bath every 10 min. We constructed concentration-response curves and determined the EC_50_.

#### Effects of *S. pilosa* aqueous extract on ileum spontaneous contractions in the presence of pharmacological blockers

In order to determine the possible mechanism of action of SpAE, a set of three experiments was conducted with a muscarinic cholinergic receptor blocker (atropine), a glutamate N-Methyl-D-aspartate (NMDA) receptor blocker (tramadol) and the standard calcium channels blocker (verapamil). Rat ileum preparations were mounted in tissue organ bath. After stabilizing the tissues for 30 min, preparations were incubated for 10 min with atropine (1 μM), tramadol (50 μM) or verapamil (0.05 μM) before administration of SpAE at the concentration of 16 mg/mL [29].

#### Effects of *S. pilosa* aqueous extract on ileum spontaneous contractions in a calcium-free Tyrode solution

The composition of the calcium-free Tyrode solution was in mM: NaCl 96.6; KCl 40; MgSO_4_ 0.8; NaHCO_3_ 11.9; NaH_2_PO_4_ 0.42; glucose 5.6 and EDTA 1 [30]. Ileum preparations were incubated with SpAE at 16 mg/mL in a Ca^2+^-free Tyrode solution containing EDTA (1 mM) in order to remove Ca^+ 2^ from the tissue and to explain the possible mechanism of action of the extract [20].

### Statistical analysis

The results are expressed as the mean ± SEM. Statistical differences between groups were evaluated with Student *t*-test or one-way ANOVA followed by Newman-Keuls multiple comparison post-test. The EC_50_ values of the extract were determined with the Variable slope model of the non-linear regression analysis. Analyses were performed using GraphPad Prism version 5.04 (GraphPad Software, San Diego, CA, USA). Differences were considered significant at *p* < 0.05.

## Results

### Effect of *Sida pilosa* aqueous extract on the body weight and relative organs weights

The body weight of uninfected-untreated mice (group UIC), infected-untreated mice (group IC), as well as infected-treated mice with praziquantel (group PZQ) or *S. pilosa* aqueous extract (SpAE 200) significantly increased (*p* < 0.001) at the end of the treatment compared to their body weight before the treatment. However, the weight gain of infected-untreated mice was 40% lower than the one of uninfected-untreated mice (*p* < 0.05). No statistical difference was recorded between the weight gain of infected-treated mice (groups PZQ and SpAE 200) and that of healthy mice (group UIC) (Table 1).

**Table 1:** Effect of oral treatment of *Sida pilosa* aqueous extract on the body weight and organs weights of *Schistosoma mansoni*-infected mice

Groups	Body weight (g)		Body weight variation (%)	Liver weight (g/100 g BW)	Intestine weight (g/100 g BW)	Spleen weight (g/100 g BW)
	Pre-infection / Pre-treatment	Post-infection / Post - treatment				
UIC	26.03 ± 0.86	35.70 ± 0.99^$$$^	38.28 ± 4.83 (27.66–48.91)	4.65 ± 0.25	4.09 ± 0.11	0.26 ± 0.02
IC	24.91 ± 0.88	30.39 ± 0.97^$$$^	22.94 ± 3.92 (14.31–31.56)^*^	9.04 ± 0.31^***^	8.81 ± 0.32^***^	1.23 ± 0.14^***^
PZQ	24.43 ± 0.25	31.40 ± 0.46^$$$^	28.60 ± 1.77 (24.78–32.42)	4.85 ± 0.05^###^	8.39 ± 0.29^***^	0.52 ± 0.03^###^
SpAE 200	23.76 ± 0.51	29.86 ± 0.66^$$$^	26.49 ± 3.09 (19.70–33.28)	7.77 ± 0.18^***, ###, £££^	7.61 ± 0.31^***, #^	0.93 ± 0.11^***, #, ££^

*UIC* Uninfected-untreated mice, *IC* Infected-untreated mice, *PZQ* Infected mice treated with praziquantel, *SpAE 200* Infected mice treated with *Sida pilosa* aqueous extract at 200 mg/kg

Data are expressed as mean ± SEM (*n* = 6). Student *t* test was used to compare values at pre-infection / pre-treatment and values at post-infection / post-treatment: ^$$$^*p* < 0.001: values are significantly different from those at pre-infection / pre-treatment. ANOVA followed by Newman-Keuls multiple comparison test was also used for statistical analysis. ^*^p < 0.05, ^***^p < 0.001: values are significantly different from those of uninfected-untreated mice (group UIC). ^#^*p* < 0.05, ^###^*p* < 0.001: values are significantly different from those of infected-untreated mice (group IC). ^££^*p* < 0.01; ^£££^*p* < 0.001: values are significantly different from those of infected mice treated with praziquantel (group PZQ). Values in brackets represent the lower and upper 95% confidence intervals

The relative weight of the liver, the intestine, and the spleen of infected-untreated mice (group IC) significantly increased (*p* < 0.001) by 94.41%, 115.40%, and 373.08% respectively compared to that of uninfected-untreated mice (group UIC). SpAE 200 treatment succeeded in reducing the liver weight (*p* < 0.001), the intestine weight (p < 0.05) and the spleen weight (*p* < 0.05) as compared to those of infected-untreated mice. PZQ treatment induced significant reduction (*p* < 0.001) of the liver and spleen weights by 46.35% and 57.72% respectively. PZQ treatment was, in fact, more effective than SpAE 200 treatment in reducing the liver and spleen weights (Table 1).

### Effect of *Sida pilosa* aqueous extract on granuloma formation in the liver and the intestine

**Fig. 1:**
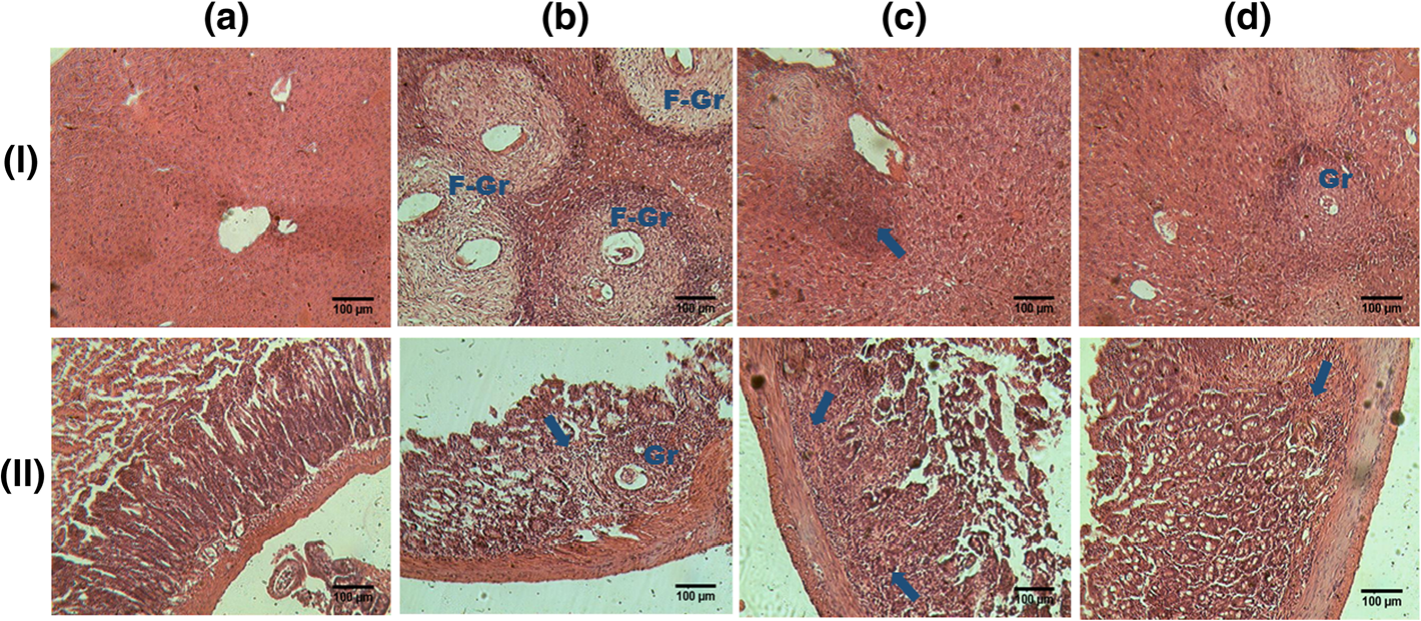
Effect of oral treatment of *Sida pilosa* aqueous extract on the liver (**I**) and ileum (**II**) histology of *Schistosoma mansoni*-infected mice. Arrows: indicate inflammatory cells infiltration; F-Gr: fibrotic granuloma; Gr: granuloma. In uninfected-untreated mice, liver (**I**-**a**) and ileum sections (**II**-**a**) showed normal structure. Multiple and voluminous granulomas surrounded with fibrous tissue were present in the liver sections of infected-untreated mice (**I**-**b**). The mucosa of their ileum contains nonfibrotic granulomas and the muscular layer was thickened (**II**-**b**). In the liver (**I**-**c**) and ileum sections (**II**-**c**) of SpAE-treated mice, scare and small granulomas were detected; while in PZQ-treated mice, liver (**I**-**d**) and ileum sections (**II**-**d**) were nearly free of granulomas

Histological examination of hematoxylin-eosin stained liver and ileum sections of uninfected-untreated mice showed normal structures (Fig. 1I-a and II-a). Ten weeks after infecting the mice with *S. mansoni*, granulomas were abundantly present around entrapped eggs in the liver. These granulomas were composed of central ova surrounded by laminated layers of collagen fibers associated with inflammatory cells at the periphery (Fig. 1I-b). Inflammatory cells were also present around congested vessels. We were able to detect non-fibrotic granulomas in the mucosa and the sub-mucosa of infected-untreated mice ileum. Compared to uninfected-untreated mice, we noticed a thickening of the *muscularis propria* of all infected mice ileum (Fig. 1II-b). The liver and the ileum of infected mice treated with praziquantel were nearly free of granulomas; only a few inflammatory cells were detected (Fig. 1I-c and II-c). After treatment of infected mice with *S. pilosa* aqueous extract, scarce and small granulomas composed of inflammatory cells were detected in the liver and in the ileum (Fig. 1I-d and II-d).

### Effect of *Sida pilosa* aqueous extract on the liver and intestine morphometry

**Fig. 2:**
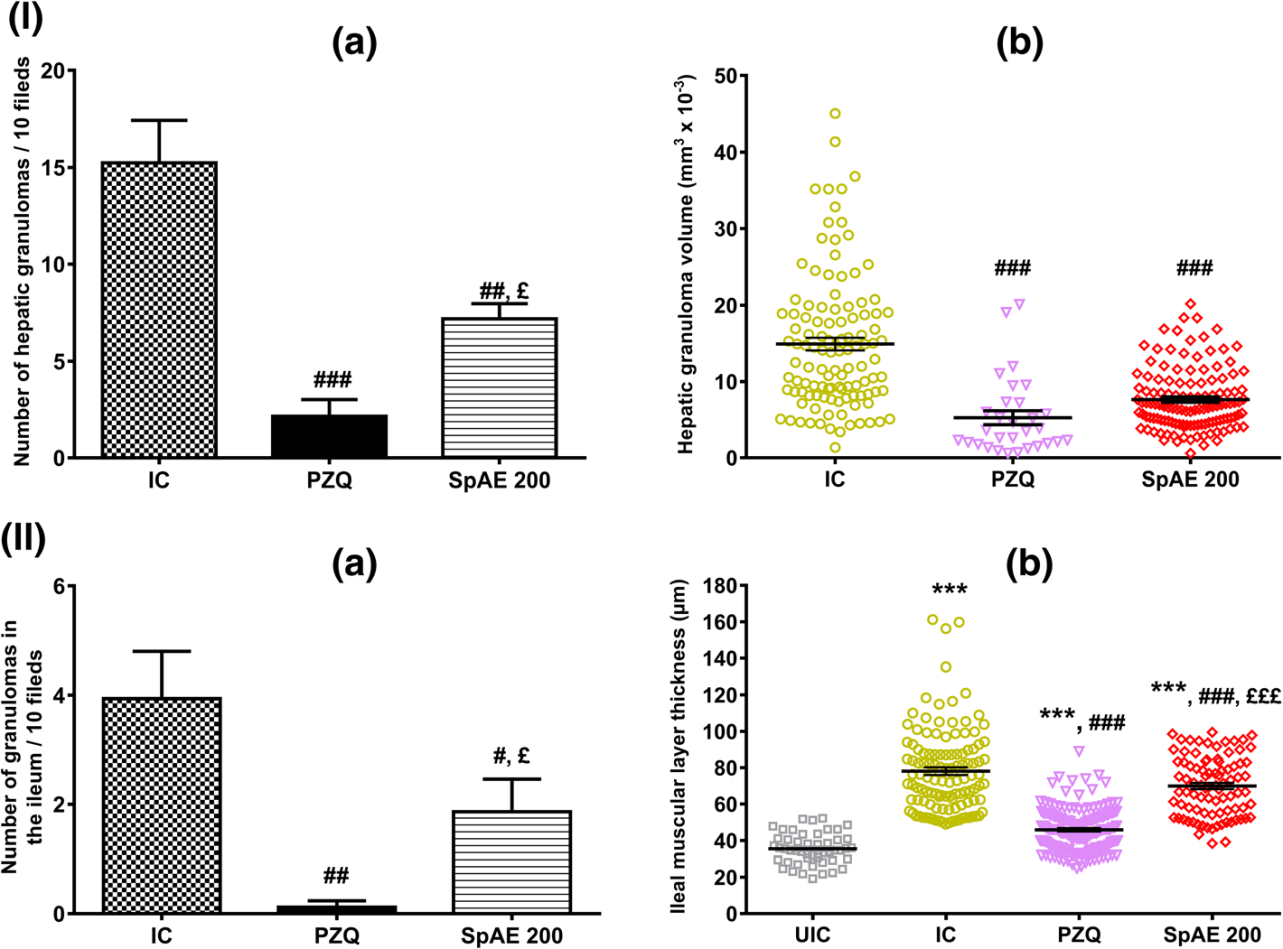
Effect of oral treatment of *Sida pilosa* aqueous extract on the morphometry of the liver (**I**) and the ileum (**II**) of *Schistosoma mansoni*-infected mice. (**a**): number of granulomas; (**b**): volume of granulomas / muscular layer thickness. UIC: uninfected-untreated mice; IC: infected-untreated mice; PZQ: infected mice treated with praziquantel; SpAE 200: infected mice treated with *Sida pilosa* aqueous extract at 200 mg/kg. Data are expressed as mean ± SEM (*n* = 6). ANOVA followed by Newman-Keuls multiple comparison test was used for statistical analysis. ^***^p < 0.001: values are significantly different from those of uninfected-untreated mice (group UIC). ^#^*p* < 0.05, ^##^*p* < 0.01, ^###^*p* < 0.001: values are significantly different from those of infected-untreated mice (group IC). ^£^*p* < 0.05, ^£££^*p* < 0.001: values are significantly different from those of infected mice treated with praziquantel (group PZQ)

Morphometric analysis of the liver showed that the granulomas number of infected-untreated mice (IC group) was 15.24 ± 2.20 and was statistically greater than that of infected-treated mice with SpAE or praziquantel. Compared to IC mice, SpAE treatment significantly reduced the granulomas number in the liver by 52.82% (7.19 ± 0.79 vs 15.24 ± 2.20) (*p* < 0.01) while the percentage of reduction after praziquantel treatment was 85.89% (2.15 ± 0.87 vs 15.24 ± 2.20) (*p* < 0.001). Praziquantel was, however, more efficient than the extract in reducing the number of granulomas in the liver (*p* < 0.05) (Fig. 2I-a). Moreover, the granuloma volume (mm^3^x10^− 3^) that was 14.91 ± 0.83 in infected-untreated mice (IC), significantly (*p* < 0.001) regressed to 7.64 ± 0.37 after SpAE treatment or to 5.26 ± 0.91 after PZQ treatment, corresponding to a reduction of 48.76% and 64.72% respectively. No statistical difference was found between SpAE and PZQ groups (Fig. 2I-b).

Compared to infected-untreated mice (IC group), SpAE administration significantly (*p* < 0.05) decreased the ileal granulomas number by 52.79%. The reduction was 96.95% after praziquantel treatment (*p *< 0.01). Praziquantel was, however, more effective than SpAE in reducing the number of granulomas in the ileum (*p* < 0.05) (Fig. 2II-a). Due to the few numbers of granulomas in the ileum, the volume of granulomas in the ileum was not evaluated. The thickness of the muscular layer of the ileum was also measured. In both infected-untreated mice (IC group) and infected-treated mice (SpAE 200 or PZQ groups), the ileal muscular layer significantly increased (*p* < 0.001) compared to that of uninfected-untreated mice (NIC group). However, SpAE or PZQ treatment successfully reduced the thickness of the muscular layer by 10.52% *(p* < 0.001) and 41.31% (*p* < 0.001) respectively compared to that of IC mice. Praziquantel was more effective than SpAE (*p* < 0.001) in reducing the ileal muscular layer thickness (Fig. 2II-b).

### Effect of *Sida pilosa* aqueous extract on total proteins and transaminases activities

The capacity of *S. pilosa* aqueous extract to improve the liver pathology induced by *S. mansoni* infection was evaluated by measuring the total proteins level and transaminases activities in the plasma. In infected-untreated mice, the concentration of total proteins significantly decreased by 13.19% (*p* < 0.05) while the activity of ALT and AST significantly (*p* < 0.001) increased by 624.32% and 135.77% respectively as compared to those of uninfected-untreated mice (Table 2). Treatment with SpAE restored the level of total proteins by significantly increasing it by 13.92% (*p* < 0.05) compared to that of infected-untreated mice. Praziquantel failed to reverse the decreasing plasma proteins concentration in infected-untreated mice. Oral administration of SpAE to *S. mansoni*-infected mice resulted in a significant decrease of ALT and AST activities by 81.09% (*p* < 0.001) and 48.93% (*p* < 0.001) respectively compared to those of infected-untreated mice. Administration of PZQ also induced a significant (*p* < 0.001) reduction by 79.63% and 32.87% of ALT and AST activities, as compared to those of IC mice. However, the reduction of AST activity induced by PZQ was not sufficient to re-establish the normal AST activity. Moreover, SpAE was as effective as PZQ in restoring ALT activity but more efficient than PZQ in restoring AST activity (Table 2).

**Table 2:** Effect of oral treatment of *Sida pilosa* aqueous extract on plasma total proteins and transaminases activities of *Schistosoma mansoni*-infected mice

Groups	Total proteins		ALT		AST	
	Concentration (mg/mL)	% change	Activity (UI/L)	% change	Activity (UI/L)	% change
UIC	0.91 ± 0.03		116.33 ± 14.28		113.59 ± 9.29	
IC	0.79 ± 0.02^*^	- 13.19	842.60 ± 91.12^***^	+ 624.32	267.81 ± 16.34^***^	+ 135.77
PZQ	0.77 ± 0.03^*^	/	171.64 ± 15.69^###^	- 79.63	179.78 ± 10.58^**, ###^	- 32.87
SpAE 200	0.90 ± 0.03^#, £^	+ 13.92	159.29 ± 16.13^###^	- 81.09	136.76 ± 9.78^###, £^	- 48.93

*UIC* Uninfected-untreated mice, *IC* Infected-untreated mice, *PZQ* Infected mice treated with praziquantel, *SpAE 200* Infected mice treated with *Sida pilosa* aqueous extract at 200 mg/kg

Data are expressed as mean ± SEM (*n* = 6). ANOVA followed by Newman-Keuls multiple comparison test was used for statistical analysis. ^*^p < 0.05, ^**^p < 0.01, ^***^p < 0.001: values are significantly different from those of uninfected-untreated mice (group UIC). ^#^*p* < 0.05, ^###^*p* < 0.001: values are significantly different from those of infected-untreated mice (group IC). ^£^*p* < 0.05: values are significantly different from those of infected mice treated with praziquantel (group PZQ)

### Effect of *Sida pilosa* aqueous extract on some liver oxidative stress biomarkers

**Fig. 3:**
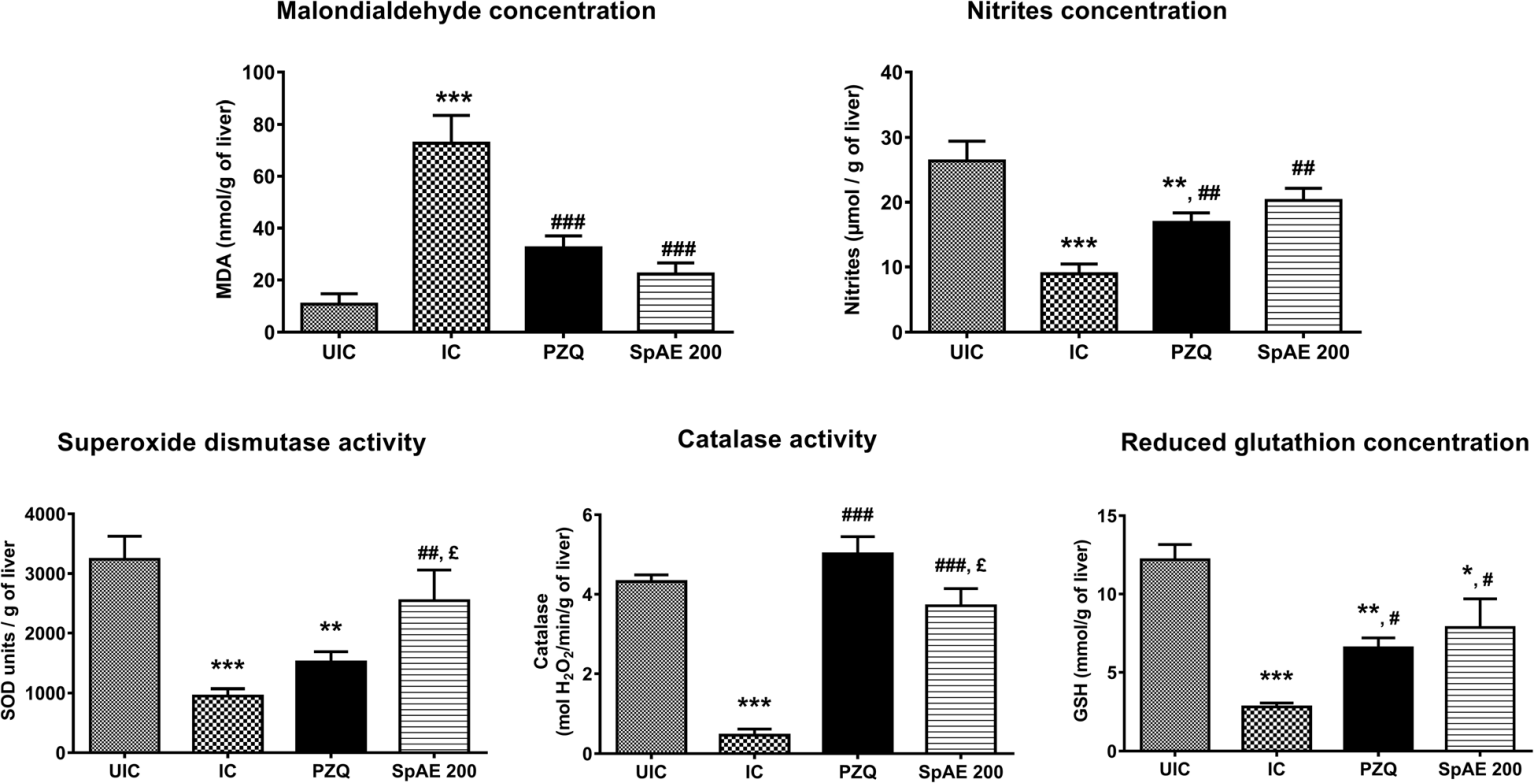
Effect of oral treatment of *Sida pilosa* aqueous extract on the levels on some hepatic oxidative stress biomarkers of *Schistosoma mansoni*-infected mice. UIC: uninfected-untreated mice; IC: infected-untreated mice; PZQ: infected mice treated with praziquantel; SpAE 200: infected mice treated with *Sida pilosa* aqueous extract at 200 mg/kg. Data are expressed as mean ± SEM (*n* = 6). ANOVA followed by Newman-Keuls multiple comparison test was used for statistical analysis. ^*^p < 0.05, ^**^p < 0.01, ^***^p < 0.001: values are significantly different from those of uninfected-untreated mice (group UIC). ^#^*p* < 0.05, ^##^*p* < 0.01, ^###^*p* < 0.001: values are significantly different from those of infected-untreated mice (group IC). ^£^*p* < 0.05: values are significantly different from those of infected mice treated with praziquantel (group PZQ)

The impact of *S. pilosa* aqueous extract treatment on the liver oxidative stress of *S. mansoni*-infected mice was also assessed in this study. *S. mansoni* infection significantly increases (*p* < 0.001) the malondialdehyde concentration which became approximately 7 fold higher than that of uninfected-untreated mice (72.69 ± 10.80 vs 10.76 ± 3.87 nmol/g of the liver). The level of nitrites was however reduced by 66.14% (*p* < 0.001) after infection. Concomitantly, the level of antioxidants was altered. Compared to those of uninfected-untreated mice, significant reductions of 70.62% superoxide dismutase (*p* < 0.001), 89.35% catalase (*p* < 0.001) and % 76.87 reduced glutathione (*p* < 0.001) were then recorded in infected-untreated mice (Fig. 3). After treating *S. mansoni*-infected mice with SpAE, we recorded a restoration of MDA, nitrites and antioxidants levels. In fact, SpAE treatment resulted in a significant decrease (*p* < 0.001) of hepatic MDA concentration by 69.33% as compared to that of infected-untreated mice. This result was similar to that obtained after praziquantel treatment. SpAE treatment normalizes the reduction of nitrites in the liver of infected mice. When compared to that of infected-untreated mice, nitrites concentration of infected mice treated with SpAE significantly increased by 126.87% (*p* < 0.01). An increase of 88.80% (*p* < 0.01) was recorded after PZQ treatment, but without restoration, since a significant difference was noted between PZQ-treated mice and uninfected-untreated mice (*p* < 0.01). SpAE treatment suppressed the *S. mansoni* infection-induced depletion of all antioxidants levels. Compared with that of infected-untreated mice, their level significantly increased in SpAE-treated mice by 167.31% (*p* < 0.01) for SOD, by 8 fold (*p* < 0.001) for catalase and by 178.72% (*p* < 0.05) for GSH. Praziquantel treatment was more effective than SpAE treatment in restoring catalase activity (*p* < 0.05) but wasn’t effective in restoring SOD activity (Fig. 3).

### Effect of *Sida pilosa* aqueous extract on the gastrointestinal transit

**Fig. 4:**
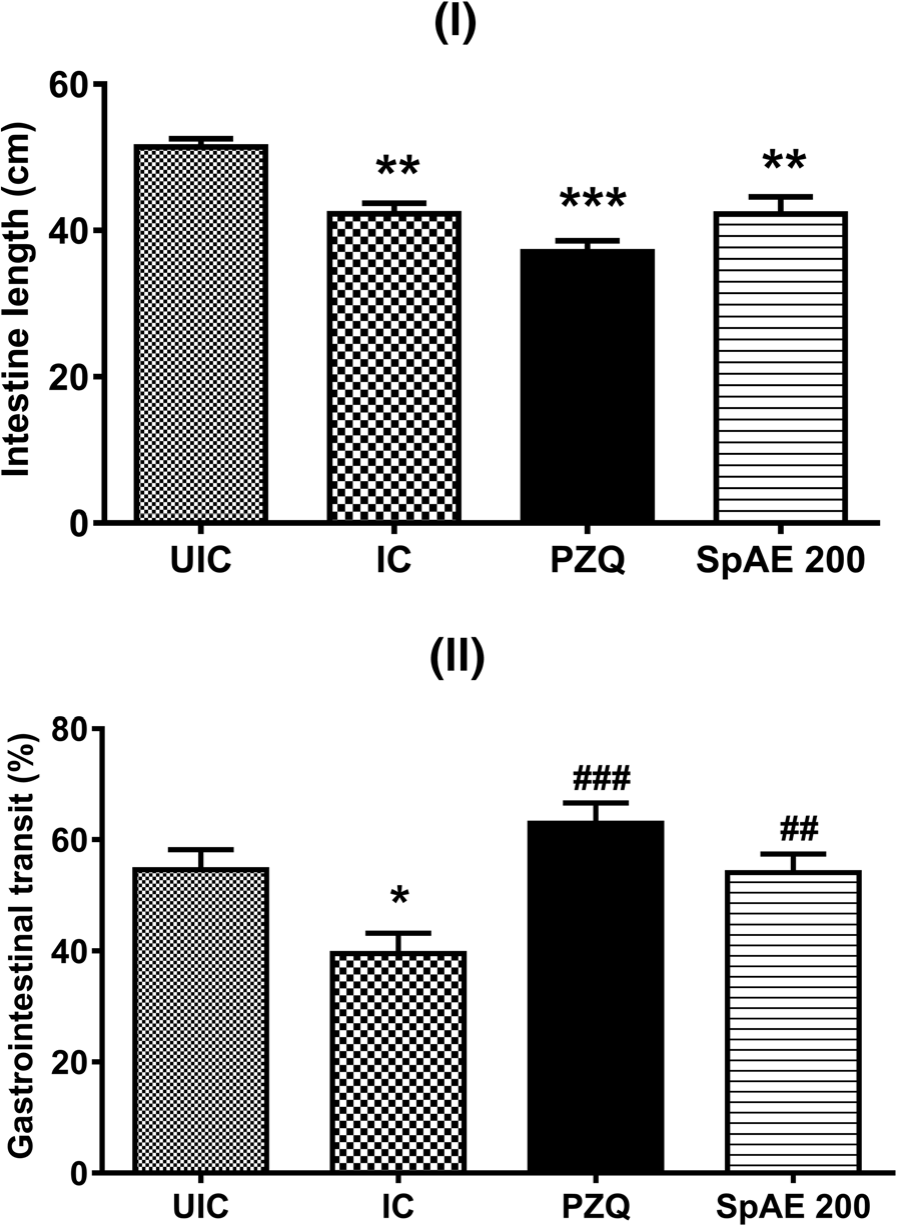
Effect of oral treatment of *Sida pilosa* aqueous extract on the intestine length (**I**) and gastrointestinal transit (**II**) of *Schistosoma mansoni*-infected mice. UIC: uninfected-untreated mice; IC: infected-untreated mice; PZQ: infected mice treated with praziquantel; SpAE 200: infected mice treated with *Sida pilosa* aqueous extract at 200 mg/kg. Data are expressed as mean ± SEM (*n* = 6). ANOVA followed by Newman-Keuls multiple comparison test was used for statistical analysis. ^*^p < 0.05, ^**^p < 0.01, ^***^p < 0.001: values are significantly different from those of uninfected-untreated mice (group UIC). ^##^*p* < 0.01, ^###^*p* < 0.001: values are significantly different from those of infected-untreated mice (group IC)

The total length of the small intestine of the control mice was 51.42 ± 1.17 cm. This was statistically higher than the length of the small intestine of infected-untreated mice (42.27 ± 1.44 cm, *p* < 0.01), infected and treated mice with praziquantel (37.07 ± 1.52 cm, *p* < 0.001) or SpAE (42.25 ± 2.36 cm, *p* < 0.01) (Fig. 4I). The gastrointestinal transit was 54.56 ± 3.60% in the control mice. After *S. mansoni* infection, the transit has significantly decreased to 39.50 ± 3.68% (*p* < 0.05) compared with control. When treated with either the plant extract or praziquantel, a normalization of the transit was observed. Compared with that of infected-untreated mice, a 36.76% (*p* < 0.01) and 59.39% (*p* < 0.001) increase of the transit was then recorded in infected-treated mice with SpAE or praziquantel respectively (Fig. 4II).

### Effect of *Sida pilosa* aqueous extract on the ileum spontaneous contractions

**Fig. 5:**
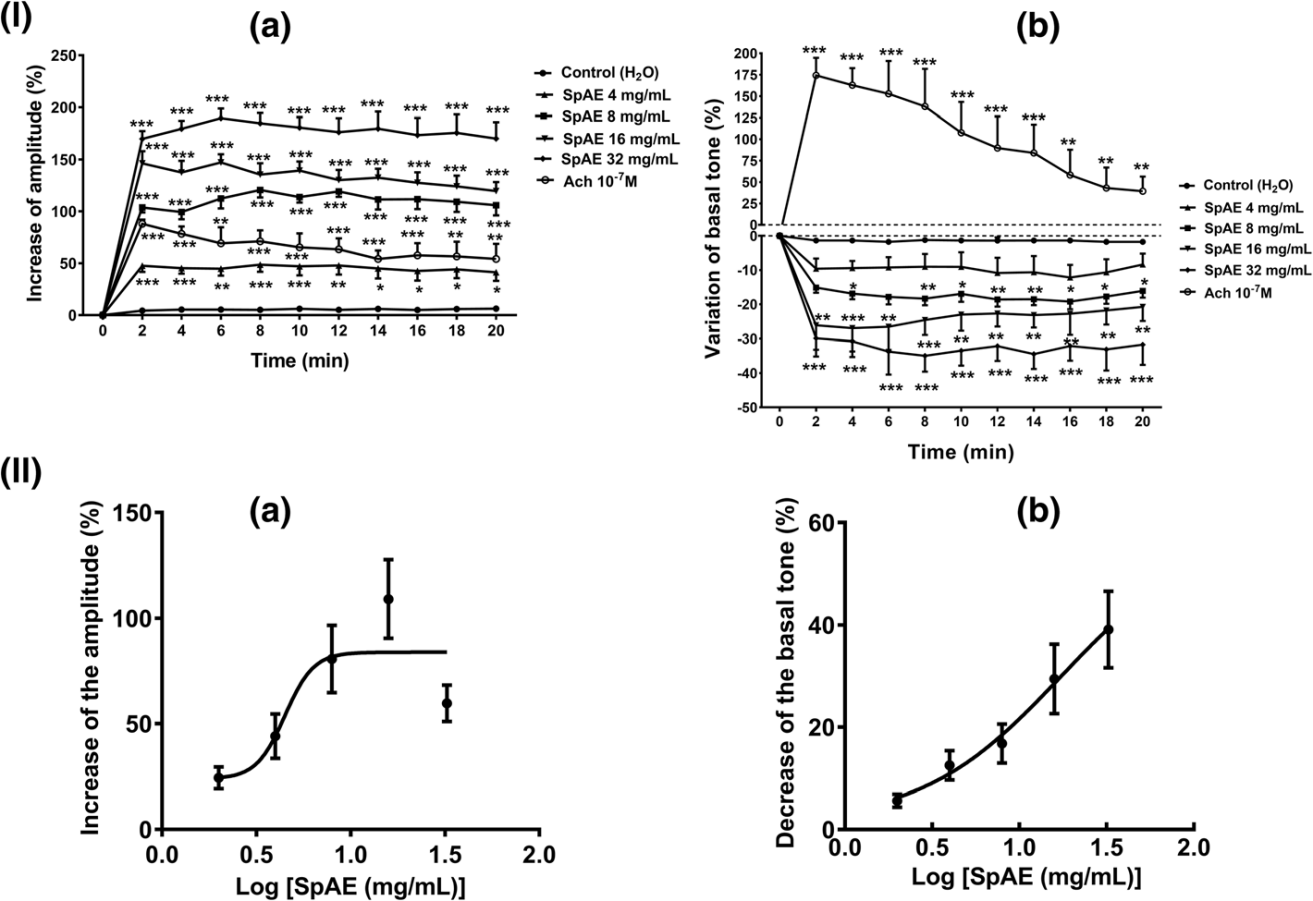
Time-response curves (**I**) and concentration-response curves (**II**) of the effect of *Sida pilosa* aqueous extract alone or in the presence of pharmacological blockers on the amplitude (**a**) and the basal tone (**b**) of spontaneous contractions of isolated rat ileum. To evaluate the time-response effect of SpAE, it was separately administered (4, 8, 16 and 32 mg/mL) to the bath solution and the effect observed on spontaneous contractions for 20 min. The vehicle was administered at equivalent volume to that of the maximal concentration of extract. The percentages of variation of the tonus and the amplitude of spontaneous contractions were calculated considering the baseline values (before drug administration) as not changes (0%). To evaluate the concentration-response effect of SpAE, it was cumulatively administered (2, 4, 8, 16 and 32 mg/mL) to the bath solution at 10 min intervals. Each point represents the mean ± SEM (*n* = 5). ANOVA followed by Newman-Keuls multiple comparison test was used for statistical analysis. ^*^p < 0.05, ^**^p < 0.01, ^***^p < 0.001: values are significantly different from those recorded with the control (H_2_O) at each time point

Rat ileum suspended in Tyrode’s solution displayed spontaneous phasic contractions with an amplitude of 0.16 ± 0.01 g, a basal tone of 3 ± 0.1 g and a frequency of 27,07 ± 0,88 contractions /min. *S. pilosa* aqueous extract (SpAE: 4, 8, 16 and 32 mg/mL) in single administration significantly increased the spontaneous contractions in a concentration-dependent manner by increasing the amplitude of the contractions (*p* < 0.001) by 48.59 ± 6.83%, 120.62 ± 7.26%, 147.10 ± 7.93% and 189.61 ± 9.16% at 4, 8, 16 and 32 mg/mL respectively compared to that of the control. Acetylcholine (10^− 7^ M) produced a significant (*p* < 0.01) increase of the amplitude of contractions which was however 40.16% lesser than the increase produced by SpAE at 16 mg/mL (Fig. 5I-a). Administered cumulatively, SpAE (2–16 mg/mL) increased the amplitude of spontaneous contractions of the rat ileum in a concentration-dependent manner. The maximal effect of SpAE was achieved at 16 mg/mL. However, the highest concentration (32 mg/mL) induced after 3 min a decrease of the amplitude of contractions. The concentration of SpAE which induced 50% of the maximal amplitude of contractions (EC_50_) was 4.49 mg/mL (Fig. 5II-a). The basal tone in the contrary slightly decreased (*p *< 0.01) by 19.19 ± 2.24%, 26.95 ± 6.75% and 34.94 ± 4.64% after SpAE administration at 8, 16 and 32 mg/mL respectively compared to that of the control. Administration of acetylcholine (10^− 7^ M) induced an immediate and significant (*p* < 0.001) increase of the basal tone by 174.30%, followed by a progressive decrease (Fig. 5I-b). Administered cumulatively, SpAE (2–32 mg/mL) decreased the tonus of spontaneous contractions of the rat ileum in a concentration-dependent manner. The concentration of SpAE capable of reducing the basal tone by 50% (EC_50_) was 17.35 mg/mL (Fig. 5II-b). SpAE concentrations of 4 and 8 mg/mL did not modify the frequency of contractions of the ileum, but at 16 and 32 mg/mL, a significant (*p* < 0.05) decrease of the frequency of contractions by 16.91% and 16.18% respectively was recorded.

### Effect of pharmacological blockers on the amplitude of ileum spontaneous contractions

**Fig. 6:**
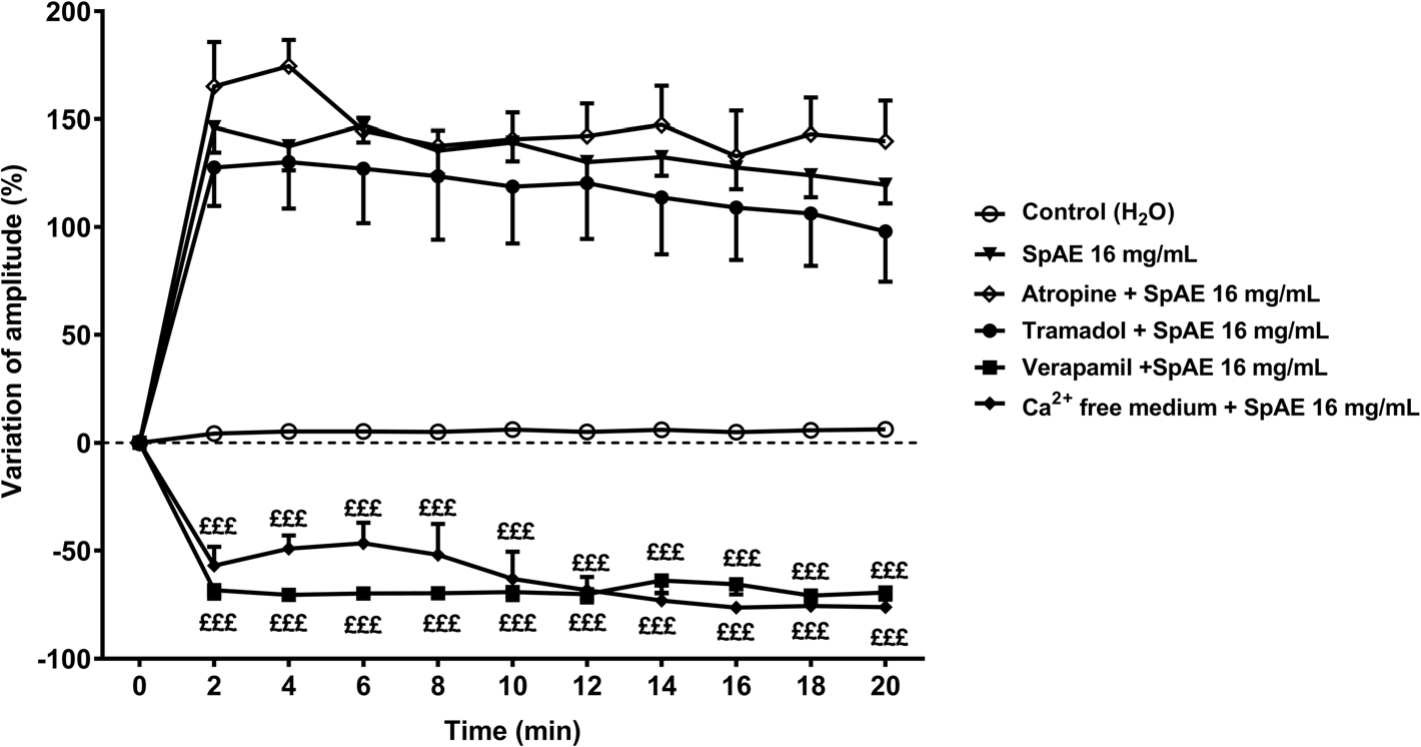
Time-response curves of the effect of *Sida pilosa* aqueous extract and pharmacological blockers on the amplitude of spontaneous contractions of isolated rat ileum. Each point represents the mean ± SEM *(n* = 5). ANOVA followed by Newman-Keuls multiple comparison test was used for statistical analysis. ^£££^*p* < 0.001: values are significantly different from those recorded with SpAE 16 mg/mL at each time point

To assess the mechanisms underlying the contractile action of *S. pilosa* aqueous extract (SpAE) on ileum, we carried out an evaluation of its effect in tissues pre-treated with different pharmacological blockers. Pre-treatment of ileum preparations with the muscarinic cholinergic receptor blocker atropine (1 μM) or the glutamate NMDA receptor blocker tramadol (50 μM) did neither altered, nor increased the amplitude of contractions, nor decreased the basal tone elicited by SpAE at 16 mg/mL. In fact, in the presence of atropine 1 μM or tramadol 50 μM, the amplitude of contractions induced by SpAE at 16 mg/mL was 174.49 ± 12.26% and 130.14 ± 21.59% respectively compared with that of the control. These results were not statistically different from the 147.10 ± 7.93% increase of amplitude induced by SpAE 16 mg/mL in the absence of atropine or tramadol. The calcium channels blocker verapamil (0.05 μM) decreased the amplitude of spontaneous contractions by 72.18 ± 2.24% and the basal tone by 145.89 ± 36.63% compared with those of the control. When ileum preparations were pre-treated with verapamil 0.05 μM, the amplitude of spontaneous contractions induced by SpAE 16 mg/mL has decreased by 70.90 ± 3.93% compared with that of the control. This amplitude was significantly (*p* < 0.001) reduced by158.80% compared to that induced by SpAE in the absence of verapamil. Likewise, the amplitude of the ileum spontaneous contractions decreased significantly by 76.71 ± 2.70% and 76.54 ± 2.89% in a free-calcium medium + EDTA 1 mM solution and in a free-calcium medium + EDTA 1 mM with SpAE 16 mg/mL respectively, as compared with that of the control. The amplitude of contractions in a free-calcium medium + EDTA 1 mM was 163.79% lower than the amplitude induced by SpAE 16 mg/mL in a normal Tyrode medium (Fig. 6).

## Discussion

*Sida pilosa* aqueous extract (SpAE) have been shown to be effective against *Schistosoma mansoni* infection in the murine model through the reduction of hepatosplenomegaly, worm burden and egg burden in the feces and the liver [16]. Most recently, antioxidant, anti-inflammatory and anti-fibrotic activities of SpAE in *S. mansoni*-infected mice were demonstrated through the evaluation of some liver biomarkers [7]. Based on histology supplemented with morphometry, this study has assessed the effect of SpAE on the granulomatous inflammation induced by *S. mansoni* infection in the liver and the intestine. Gastrointestinal motor activity was also evaluated. We demonstrated the presence of granulomas surrounding entrapped *S. mansoni* eggs in the liver of mice after 10 weeks of infection. These granulomas consisted mainly of collagen fibers and scarce inflammatory cells. In the ileum, mucosal inflammation and granulomas consisted mainly of macrophages and eosinophils surrounded by lymphocytes were present. An increase of the ileal muscular layer thickness was also observed. This was linked to the infiltration of inflammatory cells in the mucosa and submucosa. Described by Lenzi et al. [31] as an organoid, dynamic and hybrid structure formed by eosinophils, neutrophils, lymphocytes, macrophages, epithelioid cells, giant cells, mast cells, reticular fibres and fibroblasts surrounding schistosome eggs trapped in an organ, the schistosomal granuloma is commonly present in the liver, the intestine, the spleen and the lung of *S.mansoni*-infected animals [1, 2, 13, 21, 32]. The increase of intestinal wall thickness after *S. mansoni* infection has also been described in previous studies [1, 2, 11].

In the current study, morphometric analyses of the liver and the ileum demonstrated that *S. mansoni* infection considerably increases the number of granulomas in the liver and the intestine, their volume in the liver, as well as the ileal muscular layer thickness. The presence of numerous and voluminous granulomas in the liver and the intestine of *S. mansoni*-infected mice probably explain the enlargement of these organs. The regression of hepatosplenomegaly and intestine enlargement after SpAE treatment could follow the reduction of *S. mansoni* egg load in the liver, the spleen, and the intestine. Jatsa et al. [7, 16] have in fact demonstrated the schistosomicidal activity of SpAE through the reduction of the liver and intestine egg burden. Since the inflammatory reaction leading to the formation of a granuloma is initiated by schistosome eggs trapped in organs as liver and intestine, the consequence of the reduction of egg load in those organs could be the reduction of the recruitment and migration of inflammatory cells around schistosome eggs, and therefore the granulomatous reaction. In *S. mansoni* infection, if granuloma formation could be prevented or suppressed, the development of severe disease might be averted [13]. Data from the present study showed that treatment of *S. mansoni*-infected mice with SpAE significantly reduces granuloma number in the liver and the ileum by 52.82% and 52.79% respectively, as well as granuloma volume in the liver by 48.76%. The thickness of the ileal muscular layer was slightly but significantly reduced by 10.52%. Reduction of granuloma number and/or size has been previously reported by others authors after treating *S. mansoni*-infected mice with curcumin [13], *Zingiber officinale* rhizomes [33], berberine [4], *Ceratonia siliqua* pod [8], selenium nanoparticles [5] or vaccination of mice with the tegumental antigen of *S. mansoni* adult worms prior to infection [34]. The formation and the development of hepatic granuloma are intimately linked to inflammatory cells infiltration in the liver. The reduction of these cells infiltration would, therefore, induce a reduction of hepatic granuloma. It has been proved that SpAE reduces eosinophils’ and neutrophils’ infiltration through reduction of eosinophil peroxidase and myeloperoxidase activities [7]. The reduction of the granuloma number and volume could also be attributed to the anti-inflammatory and antifibrotic properties of SpAE as recently demonstrated in a murine model of *S. mansoni* infection [7]. In this study, SpAE exacerbated anti-inflammatory activity through the restoration of ALT and AST activities in infected mice. ALT and AST are biomarkers of the integrity of hepatocytes. In *S. mansoni*-infected mice, deposition of schistosome eggs in the liver induces granulomatous lesions which leads to the release of transaminases from the injured hepatic cells to the bloodstream. This granulomatous reaction is also associated with the generation of reactive oxygen species and the impairment of antioxidant defense [7, 8, 13]. The reduction of the granuloma number and volume in the liver of infected-treated mice could be correlated to the normalization of transaminases activity in these mice. The antioxidant potential of SpAE could also contribute to the reduction of the granulomatous reaction. Results from this study shown that SpAE displayed significant antioxidant activity through the reduction of malondialdehyde concentration and the activation of antioxidants such as catalase, superoxide dismutase, and reduced glutathione. The in vitro and in vivo antioxidant potential of SpAE has been previously demonstrated and was correlated to the high content of total phenolic compounds of this extract [7, 17]. Since reactive oxygen species act as both signaling molecules and mediators of inflammation, the reduction of their concentration in the liver will be in favor of the reduction of granulomatous inflammation.

During *S. mansoni* infection, morphological alterations of the ileum might affect intestinal smooth muscle function, leading to a dysfunction of the gastrointestinal transit [1, 2, 12]. In this study, when granulomas were present in the ileum of *S. mansoni*-infected mice, a significant decrease of the small intestine length and a significant reduction of the gastrointestinal transit were recorded. The current study, in line with findings of previous reports [1, 35] indicates that during *S. mansoni* infection, gastrointestinal motility is substantially altered. The presence of mucosal inflammation of the ileum probably inhibits the propulsive activity of the normal gut, leading to a dysfunction of the intestinal peristalsis and thus a decrease of the gastrointestinal transit [1]. Abdu [12] has reported a significant variation of peristaltic pressure waves amplitude and intervals in a murine model of *S. mansoni* infection. Clinical manifestations of intestinal schistosomiasis such as constipation could be the consequence of a dysfunction of the gastrointestinal motor system [9, 10]. A normal gastrointestinal transit was however restored after the treatment of *S. mansoni*-infected mice with SpAE which produced a prokinetic effect. This result could be attributed to the reduction of the granulomatous inflammation of the ileum which consequently suppresses the inhibition of the intestinal peristalsis and thus of the gastrointestinal transit. An increase of the gastrointestinal transit has been reported in the literature, where the flavonoid isoliquiritigenin isolated from the roots of *Glycyrrhiza glabra* and *Terminalia chebula* displayed contractile effect on isolated rat stomach fundus or ileum [20, 36]. Another explanation for the restoration of the gastrointestinal transit could be the increase of the contractile activity of ileal muscles by SpAE. To verify this hypothesis, the effect of SpAE on the contractile activity of the ileum was studied. *Sida pilosa* aqueous extract (SpAE) elicited a spasmogenic effect on the rat ileum by significantly increased, on a concentration-dependent manner the amplitude of spontaneous contractions (EC_50_ = 4.49 mg/mL), but decreased the basal tone of spontaneous contractions (EC_50_ = 17.35 mg/mL). In previous studies, the aqueous and ethanolic extracts of *Sida veronicaefolia* (syn. *Sida pilosa*), as well as sidaverin derived from *S. veronicaefolia*, have been shown to exhibit a spasmogenic effect on ileal and uterine smooth muscle [18, 19].

The gastrointestinal smooth muscles are spontaneously active with the periodic generation of slow waves whom the activity is initiated by the interstitial cells of Cajal distributed in the myenteric region [37]. Slow waves propagate within the interstitial cells of Cajal network, reach the smooth muscle cells via gap junctions and initiate phasic contractions by activating Ca^2+^ entry through L- type Ca^2+^ channels. The gastrointestinal motility is an integrated process modulated by local and circulating neurohumoral substances [37, 38]. In the present study, we investigated the mechanism underlying the action of SpAE on muscle contractions. The contractile effect of SpAE might involve muscarinic receptors, glutamate NMDA receptors or calcium influx into the muscle cell through voltage-gated Ca^2+^ channels. Activation of muscarinic receptors on the gastrointestinal smooth muscle cells by releasing acetylcholine from excitatory neurons plays an important physiological role in the stimulation of spontaneous muscle contractions and atropine blocks all muscarinic receptor sites. The predominant muscarinic receptor subtypes present in intestinal smooth muscles are M2 and M3, and direct contraction of the intestinal smooth muscle is mediated via the M3 subtype [29]. The failure of atropine (1 μM) to reduce the SpAE-induced contractions may indicate that the action of the extract is not through M3 muscarinic receptors. Glutamate evoked contractions of the longitudinal muscle/myenteric plexus (LMMP) preparation by acting on N-Methyl-D-aspartate (NMDA) receptors. Other agonists at the NMDA recognition site, but not quisqualate or kainate, also contracted the LMMP, and glutamate-evoked contractions are competitively inhibited by selective NMDA receptor antagonists. Evidence in the literature demonstrates that tramadol antagonizes glutamate NMDA receptors [39]. In the present study, tramadol (50 μM) did not inhibit the spasmogenic effect of SpAE on ileal muscles contractions, suggesting that the action of SpAE is not through glutamate NMDA receptors. Since it has been demonstrated that tramadol also suppresses the activity of M3 muscarinic receptors [39], the failure of tramadol to inhibit SpAE-induced contractions confirmed the non-involvement of the cholinergic system in the mechanism of action of SpAE. In smooth muscle cells, the primary stimulus for contraction is the increase in cytoplasmic calcium concentration. This increase is generally the result of both the release of intracellular Ca^2+^ from sarcoplasmic reticulum stores and the influx of extracellular Ca^2+^. The main pathway for extracellular Ca^2+^ entry into intestinal smooth muscle cells is through L-type voltage-dependent Ca^2+^ channels [28, 29, 38]. Blockade of the L-type Ca^2+^ channels using verapamil or another Ca^2+^ channel blocker abolishes or markedly reduces the contractile activity of intestinal muscle strips [29]. Results from this study showed that ileal muscle contractions induced by SpAE were drastically reduced by verapamil (0.05 μM), indicating that the action of SpAE on gastrointestinal motility is mediated by Ca^2+^ influx through L-type Ca^2+^ channels. Furthermore, SpAE-induced ileal muscle contractions were significantly reduced in a Ca^2+^-free medium with EDTA 1 mM (a calcium chelator), suggesting that SpAE mobilizes Ca^2+^ from sarcoplasmic reticulum stores.

The action of SpAE on the gastrointestinal motility is thus mediated via the mobilization of intracellular and extracellular calcium. The spasmogenic effect of SpAE may explain the prokinetic effect displayed in vivo. Chen et al. [36] have in fact demonstrated that the increase of the gastrointestinal transit of rats who have received the flavonoid isoliquiritigenin was correlated to the spasmogenic effect of this compound. The role of bioactive compounds such as alkaloids, phenols, and terpenoids in promoting contractile effect on rabbit ileum and jejunum has been demonstrated [40]. It has also been reported that tannins and flavonoids have an inhibitory effect on intestinal smooth muscles contractility [41]. The dual effect of SpAE, increasing the amplitude of spontaneous contractions but decreasing their basal tone, might, therefore, be explained by the presence of alkaloids, phenols, triterpenes, and flavonoids in SpAE [7, 16].

## Conclusion

*Sida pilosa* aqueous extract is effective in inhibiting *Schistosoma mansoni-*induced granulomatous inflammation in the liver and intestine and in re-establishing a normal gastrointestinal transit. This extract contributes to maintaining a normal gastrointestinal transit through its spasmogenic effect on the intestine by mobilizing intracellular and extracellular calcium. This study justifies the use of *Sida pilosa* aqueous extract for the treatment of schistosomiasis and others helminthiasis. It could, therefore, be used as a starting point for the development of complementary or alternative medicine against *S. mansoni* infection.

## Abbreviations

ALT: Alanine aminotransferase
ANOVA: Analysis of variance
AST: Aspartate aminotransferase
CAT: Catalase
EDTA: Ethylenediamine tetra-acetic acid
GSH: Reduced glutathione
H&E: Hematoxylin and eosin
LMMP: Longitudinal muscle/myenteric plexus
MDA: Malondialdehyde
NMDA: N-Methyl-D-aspartate
PBS: Phosphate-buffered saline
PZQ: Praziquantel
SEM: Standard error of the mean
SOD: Superoxide dismutase
SpAE: *Sida pilosa* aqueous extract
